# Prostate Cancer: Early Detection and Assessing Clinical Risk Using Deep Machine Learning of High Dimensional Peripheral Blood Flow Cytometric Phenotyping Data

**DOI:** 10.3389/fimmu.2021.786828

**Published:** 2021-12-16

**Authors:** Georgina Cosma, Stéphanie E. McArdle, Gemma A. Foulds, Simon P. Hood, Stephen Reeder, Catherine Johnson, Masood A. Khan, A. Graham Pockley

**Affiliations:** ^1^ Department of Computer Science, Loughborough University, Loughborough, United Kingdom; ^2^ John van Geest Cancer Research Centre, School of Science and Technology, Nottingham Trent University, Nottingham, United Kingdom; ^3^ Centre for Health, Ageing and Understanding Disease (CHAUD), School of Science and Technology, Nottingham Trent University, Nottingham, United Kingdom; ^4^ Department of Urology, University Hospitals of Leicester National Health Service (NHS) Trust, Leicester, United Kingdom

**Keywords:** prostate cancer, predictive modeling, immunophenotyping data, flow cytometry, PSA level, computational analysis, machine learning

## Abstract

Detecting the presence of prostate cancer (PCa) and distinguishing low- or intermediate-risk disease from high-risk disease early, and without the need for potentially unnecessary invasive biopsies remains a significant clinical challenge. The aim of this study is to determine whether the T and B cell phenotypic features which we have previously identified as being able to distinguish between benign prostate disease and PCa in asymptomatic men having Prostate-Specific Antigen (PSA) levels < 20 ng/ml can also be used to detect the presence and clinical risk of PCa in a larger cohort of patients whose PSA levels ranged between 3 and 2617 ng/ml. The peripheral blood of 130 asymptomatic men having elevated Prostate-Specific Antigen (PSA) levels was immune profiled using multiparametric whole blood flow cytometry. Of these men, 42 were subsequently diagnosed as having benign prostate disease and 88 as having PCa on biopsy-based evidence. We built a bidirectional Long Short-Term Memory Deep Neural Network (biLSTM) model for detecting the presence of PCa in men which combined the previously-identified phenotypic features (CD8^+^CD45RA^-^CD27^-^CD28^-^ (*CD8^+^ Effector Memory cells*), CD4^+^CD45RA^-^CD27^-^CD28^-^ (*CD4^+^ Effector Memory cells*), CD4^+^CD45RA^+^CD27^-^CD28^-^ (*CD4^+^ Terminally Differentiated Effector Memory Cells re-expressing CD45RA*), CD3^-^CD19^+^ (*B cells*), CD3^+^CD56^+^CD8^+^CD4^+^ (*NKT cells*) with Age. The performance of the PCa presence ‘detection’ model was: Acc: 86.79 ( ± 0.10), Sensitivity: 82.78% (± 0.15); Specificity: 95.83% (± 0.11) on the test set (test set that was not used during training and validation); AUC: 89.31% (± 0.07), ORP-FPR: 7.50% (± 0.20), ORP-TPR: 84.44% (± 0.14). A second biLSTM ‘risk’ model combined the immunophenotypic features with PSA to predict whether a patient with PCa has high-risk disease (defined by the D’Amico Risk Classification) achieved the following: Acc: 94.90% (± 6.29), Sensitivity: 92% (± 21.39); Specificity: 96.11 (± 0.00); AUC: 94.06% (± 10.69), ORP-FPR: 3.89% (± 0.00), ORP-TPR: 92% (± 21.39). The ORP-FPR for predicting the presence of PCa when combining FC+PSA was lower than that of PSA alone. This study demonstrates that AI approaches based on peripheral blood phenotyping profiles can distinguish between benign prostate disease and PCa and predict clinical risk in asymptomatic men having elevated PSA levels.

## Introduction

Currently, diagnosing prostate cancer (PCa) primarily relies on painful invasive biopsies which put ~5% of men at risk of developing life-threatening infections, such as urosepsis. As biopsy results are not definitive, there is a significant risk of misdiagnosis, over-treatment, and under-treatment. It is therefore imperative to avoid unnecessary biopsies and more accurately diagnose the presence of PCa and, if present, its clinical significance.

In a landmark study, Stamey et al. performed the first large-scale analysis of serum PSA as a prostate cancer biomarker in 1987, convincingly demonstrating that PSA was more sensitive than prostate specific acid phosphatase (PSAP)/prostatic acid phosphatase (PAP) for monitoring the disease (1). They showed that PSA levels increased with advancing clinical stage and was useful for detecting disease recurrence after curative therapy (1). In 1991, Catalona et al. demonstrated that the combination of a serum PSA measurement ≥4.0 ng/ml with other clinical findings, such as the results of a DRE, improved detection of prostate cancer in a prospective study of 1653 healthy men with no history of cancer (2).

Although the clinical introduction of the Prostate-Specific Antigen (PSA) test in 1986 increased the early diagnosis of localised PCa, elevated levels of PSA do not necessarily indicate the presence of disease, as PSA levels can be raised by prostatitis, other localised infections, benign hyperplasia and/or other factors such as physical stress. It is also the case that 15% of men with PSA levels in the normal range typically have PCa, with a further 15% of these cancers being high‐grade (https://prostatecanceruk.org/prostate-information/prostate-tests/psa-test).

Findings from a study involving 419,582 British men aged 50 to 69 years - the Cluster Randomized Trial of PSA Testing for Prostate Cancer (CAP), which was conducted at 573 primary care practices across the United Kingdom, do not support single PSA testing for population-based screening and suggest that asymptomatic men should not be routinely tested to avoid unnecessary anxiety and treatment (3). However, in contrast to the CAP study, the 16-year follow-up of the European Randomized Study of Screening for Prostate Cancer (ERSPC) which was launched in 1993 and was the world’s largest randomized controlled trial evaluating the effect of PSA screening on PCa mortality involving men aged between 50 and 69 has reported PSA screening to significantly reduce PCa-related mortality (4). Given its poor diagnostic specificity, PSA-based PCa screening is not currently supported by the UK National Health Service (NHS) or promoted in any other country.

So, how do we improve the diagnosis of PCa beyond the utilisation of PSA and digital rectal examination (DRE) alone given that measuring blood PSA levels lacks specificity and the DRE lacks both sensitivity and specificity? PSA and DRE measurements do not necessarily differentiate between clinically significant PCa, which requires treatment, and indolent cancer, for which the current recommendation is active surveillance. The challenge over the past two decades has therefore not only been to improve the diagnostic yield of PCa, but also to develop new approaches for more specifically distinguishing between benign prostate disease and PCa and, arguably more importantly, between low-risk disease which requires no treatment and clinically significant disease which requires treatment. As the diagnosis of PCa based on PSA levels and the DRE alone is not reliable, confirmation using other approaches such as invasive biopsies and/or MRI scans is required.

Traditionally, PCa has been diagnosed by performing transrectal ultrasound (TRUS) guided prostate biopsies. However, such a biopsy technique has a cancer detection rate of less than 30% in a benign feeling prostate. The major drawback in performing TRUS prostate biopsies is that it is only possible to accurately biopsy the posterior peripheral and transition zone due to limitations in mobility of the ultrasound probe. Currently, ~55% of transrectal ultrasound (TRUS) biopsies return negative results (5). A negative TRUS biopsy of the prostate does not therefore necessarily equate to a cancer-free prostate, as prostate cancer may be present in the anterior parts of the peripheral or transition zone that are inaccessible *via* such a route. As such, a negative TRUS biopsy could falsely be reassuring to the patient who then subsequently presents later with advanced/metastatic PCa. As the rectum is highly colonized with bacteria, approximately 3-5% of men who undergo TRUS guided prostate biopsies will experience potentially life threatening urosepsis (6) with many such patients requiring ITU care. Worryingly, the risk of developing urosepsis has increased over the past decade due to the development of multi-drug resistant fecal bacteria (7). Another issue is that the PCa detection rate significantly reduces when TRUS biopsies are repeated due to rising PSA (8).

The diagnostic strength of an alternative biopsy approach - the transperineal template prostate (TPTP) biopsy - which involves interrogating the entire prostate using a grid/template of needles inserted *via* the perineal skin has been shown to deliver a better rate of cancer detection than the TRUS biopsy (52%-68%) (9). Directly comparing TRUS against TPTP in biopsy naïve men has also revealed TPTP to significantly outperform TRUS with respect to the detection of PCa (60% *versus* 32%) (10). Although MRI-based diagnosis of PCa is continuing to develop, MRI cannot currently be used as a sole diagnostic to replace biopsies, as a positive MRI can be incorrect in ~25% of cases and a negative MRI incorrect in ~20% of cases. MRI can be used on patients with a PSA of 10-20 ng/ml and ~70% of these men are currently having `up front’ MRI which consumes vital healthcare resources. However, MRI does have clinical utility for staging and focusing of biopsies. It is therefore essential that misdiagnosis and unnecessary procedures are reduced by the development of non-invasive approaches such as blood tests/liquid biopsies that are more accurate at detecting and categorizing the clinical risk of PCa than the PSA test.

Given the reciprocal relationship between cancer and a patient’s immune system, we proposed, and have previously shown, that the presence of PCa is reflected by detectable changes in the peripheral blood immunome. We were the first to successfully use computational modelling of multi-parametric flow cytometry data of peripheral blood T and B cells to identify phenotypic profiles (‘signatures’) which, when input into a computational machine learning tool, reliably identifies the presence of PCa in asymptomatic men with PSA levels <20 ng/ml (11). Managing this group of individuals presents a particularly significant clinical quandary, because although only 30%-40% of these men will have PCa, currently all must undergo potentially unnecessary invasive prostate biopsies. For this study (11) we devised a combinatorial feature selection method to identify a unique peripheral blood immune cell phenotypic profile (`signature’) of five T and B cell phenotypic ‘features’ which was incorporated into an interpretable machine learning model. Our approach achieved 83% accuracy, *versus* 77.78% for the PSA test, and decreased false positives by 12.9% (11).

Using samples from the same cohort of asymptomatic men having PSA levels <20 ng/ml, we subsequently demonstrated that incorporating eight peripheral blood natural killer (NK) cell phenotypic features into an Ensemble machine learning prediction model could also distinguish between the presence of benign prostate disease and PCa. Furthermore, and very importantly, we could also demonstrate that the machine learning model, when adapted to incorporate 32 NK cell phenotypic features, could predict the D’Amico Risk Classification (clinical risk of PCa) in those patients identified as having PCa and was thereby able to accurately differentiate between the presence of low-/intermediate-risk disease and high-risk disease without the need for additional clinical data (12). These studies used Genetic Algorithms to identify combinations of phenotypic features which were used to develop prediction models based on the k-Nearest Neighbour (kNN) classification algorithm and Ensemble machine learning prediction models (11, 12).

The phenotypic datasets utilised in our previous studies were generated from asymptomatic men who had PSA levels <20 ng/ml and who had all undergone diagnosis using the more definitive TPTP biopsy (11, 12). The aim of the study presented herein is to extend the findings of our previous studies to determine whether the T and B cell phenotypic features which have previously been identified as being able to distinguish between benign prostate disease and PCa in asymptomatic men having PSA levels < 20 ng/ml (11) can also be used to detect the presence and clinical risk of PCa in a larger cohort of patients whose PSA levels ranged between 3 and 2617 ng/ml, the PCa disease status of whom had been determined using either the TPTP or TRUS biopsy. For this, we implemented two separate bidirectional Long Short-Term Memory Deep Neural Network (biLSTM) models, one for predicting the presence of PCa and another for predicting the clinical risk of any PCa present, as defined by the D’Amico Risk Classification. Given limited sample numbers, it was not possible to undertake a similar analysis using the NK cell phenotyping dataset from our previous study (12).

## Materials and Methods

### Sample Collection

Peripheral blood samples were obtained from asymptomatic men suspected of having PCa that attended the Urology Clinic at Leicester General Hospital (Leicester, UK) between 24 October 2012 and 15 August 2014. Samples were obtained from two cohorts of patients, termed the ‘TPTP’ and ‘TRUS’ cohorts (see below for more details). For both cohorts, patients were recruited and treated as described previously (10).

### Data Collection

Phenotypic data were generated from a total of 130 males (42 diagnosed with benign disease and 88 diagnosed with cancer, as confirmed by TPTP or TRUS biopsy evidence) (**Tables 1**, **2**). Of the 42 subjects diagnosed with benign disease; 2 were diagnosed with Atypical Small Acinar Proliferation (ASAP). 11 with Atypia, 13 with High Grade Prostatic Intraepithelial Neoplasia (PIN) and 16 with benign disease. Of the men diagnosed with PCa, 18 had low-risk, 44 had intermediate-risk, and 25 had high-risk cancer based on their D’Amico Risk Classification for Prostate Cancer (13). The D’Amico Risk for one patient was not available as no Gleason score values were provided. Further details regarding the TRUS (14) and TPTP (9, 10) biopsy techniques have been provided elsewhere.

**Table 1:** Clinical demographics of cohorts.

	No. of Patients	Min. Age	Max. Age	Mean Age	SD. Age	Min. PSA	Max. PSA	Mean PSA	SD. PSA
**Total: Benign**	42	51	77	65.88	5.76	4.70	19.00	8.43	3.33
**ASAP**	2	60	61	60.50	0.50	5.30	7.80	6.55	1.25
** Atypia**	11	51	77	64.73	7.19	4.70	19.00	8.23	3.71
** High PIN**	13	54	75	64.46	5.93	5.10	12.00	7.82	2.27
** Benign**	16	63	5.3	68.50	3.16	5.30	18.00	9.29	3.70
**Total Cancer:**	88	52	88	69.88	7.97	3.00	2617.00	51.00	277.41
** Low**	18	55	78	65.33	5.92	4.70	9.80	6.55	1.55
** Intermediate**	44	53	88	69.75	8.04	3.00	19.00	9.56	3.49
** High**	25	52	88	73.36	7.59	4.30	2617.00	157.10	505.11
** Unknown**	1	70				19.00			

**Table 2:** Clinical demographics of TRUS and TPTP biopsy cohorts.

TRUS Biopsy Cohort					
TRUS Gleason grade	TRUS Gleason score	Number of patients	Group Age Range (yr)	Group PSA range (ng/ml)	Clinical RIsk
Benign	Benign	6	63-75	5.5-18	–
	High PIN	3	60-69	6.9-12	–
	Atypia	2	54-67	4.9-5.6	–
Gleason 6	3+3	13	55-88	3.9-19	Low-Intermediate
Gleason 7	3+4	10	52-86	3-76	Intermediate - High
	4+3	8	63-85	7.8-248	Intermediate - High
Gleason 8	4+4	2	70-74	7.9-12	High
Gleason 9	4+5	9	67-88	4.3-2617	High
	5+4	2	69-84	40-118	High
Unknown	Cancer	1	74	75	High
Small cell	Cancer	2	66-80	59-83	High
**TPTP Biopsy Cohort**					
**TPTP Gleason Grade**	**TPTP Gleason score**	**Number of patients**	**Group Age range (yr)**	**Group PSA range (ng/ml)**	**Stage**
Benign	Benign	10	65-71	5.3-15	–
	High PIN	10	54-71	5.1-12	–
	ASAP	2	60-61	5-3-7.8	–
	Atypia	9	51-77	4.7-19	–
Gleason 6	3+3	16	56-85	4.7-11	Low-Intermediate
Gleason 7	3+4	18	53-79	4.7-13	Intermediate
	4+3	5	55-81	5.1-19	Intermediate
Gleason 9	4+5	2	70-75	6.3-18	Intermediate

Some of the data used in the present study have been previously published (11). These data were derived from 72 males having PSA levels < 20 ng/ml who had a TRUS and then a TPTP biopsy (11). The mean age for this cohort was 66 years old (age range of 50–84 years old).

### Flow Cytometric Analysis

Peripheral blood was collected from all individuals using standard clinical procedures, aliquots of which (30 ml) were transferred into sterile 50 ml polypropylene (Falcon) tubes containing 300 µl sterilised Lithium Heparin (1000 U/ml, Merck Millipore). Anti-coagulated samples were immediately transferred to the John van Geest Cancer Research Centre at Nottingham Trent University (Nottingham, UK) and processed immediately upon receipt (always within 3 hours of collection).

**Figure 1:**
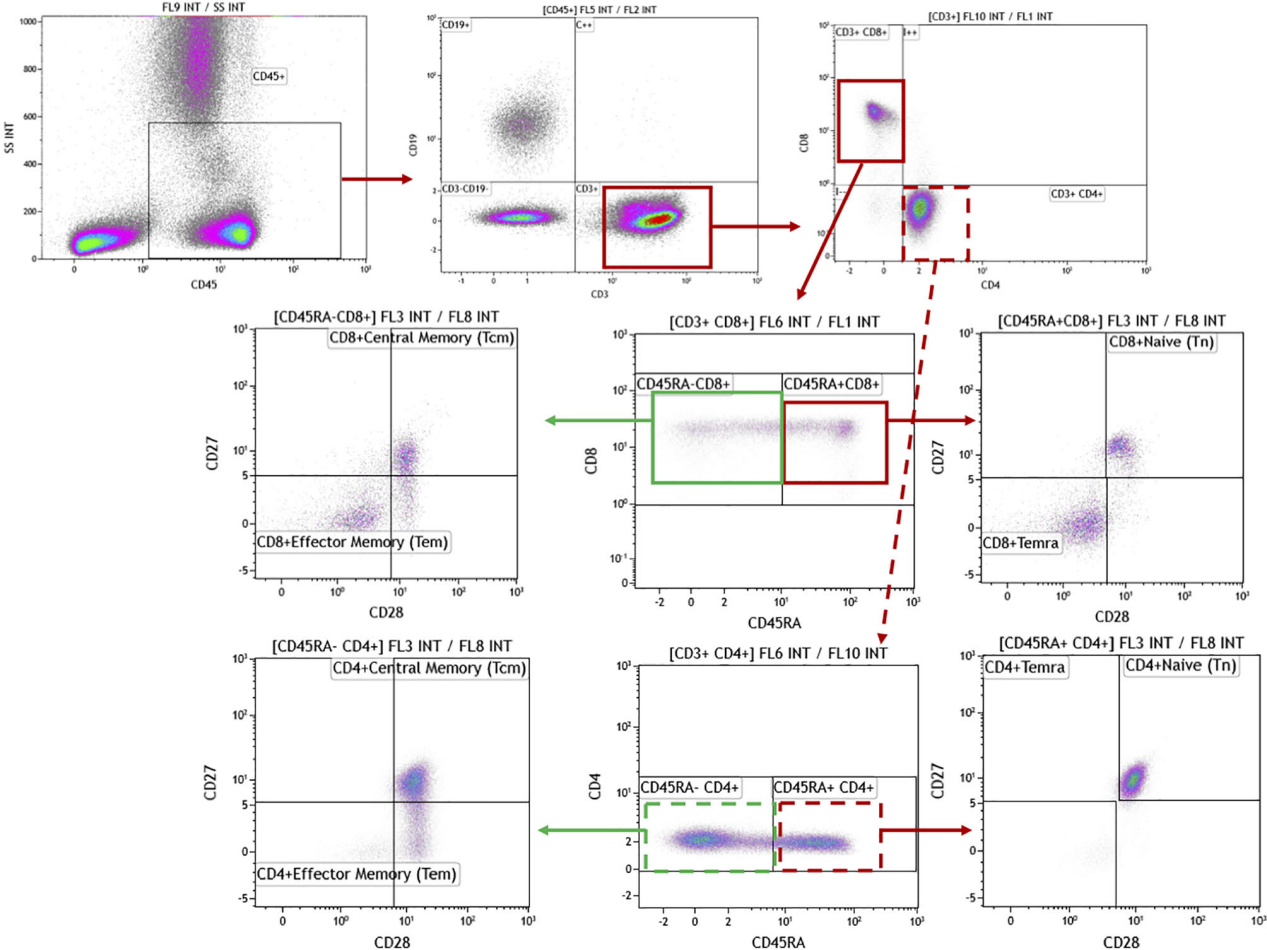
Representative gating strategies for the flow cytometric analysis of single cells. The staining panel confirmed CD45 expression then determined cell populations as CD14^+^ monocytes, CD3^-^CD56^+^ NK cells (with CD56^bright^ and CD56^dim^ subsets), CD3^+^CD56^+^ NKT cell subpopulations, CD19^+^ B cells, CD3^+^CD4^+^ and CD3^+^CD8^+^ Naïve, Central Memory, Effector Memory, Terminally Differentiated Effector Memory Cells Expressing CD45RA T cell populations. The definition of monocytes based on CD45^+^CD4^+^ generated the same data as defining them based on CD3^-^CD14^+^ (data not shown).

Absolute cell counts in whole blood samples were determined by the inclusion of BD Trucount™ beads (BD Biosciences; Mountain View, CA, USA), as per the manufacturer’s protocol. For the flow cytometric analysis, 100 μl of blood was mixed directly in the BD Trucount™ bead tube and T cell, B cell, and NK cell populations identified using the conjugated monoclonal antibodies (mAbs) detailed in **Table 3**. Samples were incubated for 15 min at room temperature, protected from the light, after which erythrocytes were lysed by incubating samples for 15 min at room temperature in BD Pharm Lyse™ (BD Biosciences). Once staining was complete, cells were washed in phosphate buffered saline (PBS), resuspended in Coulter Isoton™ diluent. Data were acquired within 1 h using a 10-color/3-laser Beckman Coulter Gallios™ flow cytometer and analyzed using Kaluza™ v1.3 data acquisition and analysis software (Beckman Coulter). Controls used a “Fluorescence minus One”, “FMO” approach (15). A typical gating strategy for the analyses is presented in **Figure 1**.

**Table 3:** Monoclonal antibody (mAb) panel for B and T cell phenotyping.

Antibody	Fluorochrome	Clone	Supplier
CD8	FITC	SK1	BioLegend
CD19	PE	HIB19	BioLegend
CD28	PE-Texas Red (ECD)	CD28.2	Beckman Coulter
CD56	PE-Cy5™	NCAM	BioLegend
CD3	PE-Cy7™	HIT3a	BioLegend
CD45RA	Allophycocyanin (APC)	HI100	eBioscience
CD14	Alexa Fluor™ 700	HCD14	BioLegend
CD27	APC eFluor™ 780	O323	eBioscience
CD45	Pacific Blue™	J33	Beckman Coulter
CD4	Krome Orange	13B8.2	Beckman Coulter

### Deep Neural Networks for Predicting the Presence of PCa and Its Clinical Risk (biLSTM)

The bidirectional Long Short-Term Memory Deep Neural Network (biLSTM) is also known as a bidirectional Recurrent Neural Network (RNN). LSTM is an artificial recurrent neural network architecture used in the field of deep learning. Unlike the standard feedforward neural network, the LSTM has feedback connections which enable it to process entire sequences of data. A biLSTM is a type of LSTM with a bidirectional layer and learns bidirectional long-term dependencies in sequence data. The architecture of the proposed biLSTM for detecting the presence of PCa is shown in **Table 4**. Although the biLSTM is widely applied to sequential data, it has been, and can also be successfully applied to non-sequential data.

**Table 4:** Parameter settings of the Deep Learning Models.

maxEpochs	20
miniBatchSize	10
Initial learning rate	0.01
Shuffle	Every epoch
**BiLSTM layers**	
Sequence Input	Sequence input with n dimensions, where n is the number of features
BiLSTM	BiLSTM with 256 hidden units
Fully Connected	2 fully connected layer
Fully Connected	3 fully connected layer
Softmax	Softmax
Classification Output	crossentropyex
Solver	Stochastic gradient descent with momentum (SGDM) optimizer

A biLSTM model learns the input sequence both forward and backwards and concatenates both interpretations. The model duplicates the first recurrent layer in the network and creates two side-by-side layers, then provides the input sequence ‘as-is’ as input to the first layer and providing a reversed copy of the input sequence to the second (16). The training data are shuffled before each training epoch, and the validation data are shuffled before each network validation. Given that the mini-batch size does not evenly divide the number of training samples, the network discards the training data that do not fit into the final complete mini-batch of each epoch. Shuffling the data as mentioned above avoids discarding the same data at every epoch.

Two biLSTM models were implemented. The first biLSTM model takes as input immunophenotypic features and clinical data and is trained to detect the presence of PCa. The second model takes as input a set of biomarkers comprising immunophenotypic features and clinical data and is trained to predict the clinical risk the PCa when PCA has been identified as being present.

**Figure 2:**
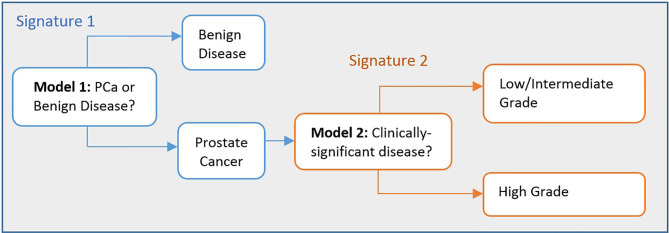
Flow chart illustrating the process to detect the presence of PCa and its clinical significance. Stage 1 (Model 1): distinguishes between men with benign prostate disease and PCa; Stage 2 (Model 2): predicts risk (in terms of clinical significance) in men identified as having PCa in Stage 1. Note that Stage 1 can also detect PCa in men with PSA levels < 20 ng/ml.

The models were built using combinations of phenotypic features and clinical data to determine the best combinations for training each model. **Figure 2** shows how prediction models for detecting the presence of PCA and its clinical risk can be utilised to assist clinical diagnosis.

### Methodology for Evaluating the Deep Neural Network Models

**Figure 3:**
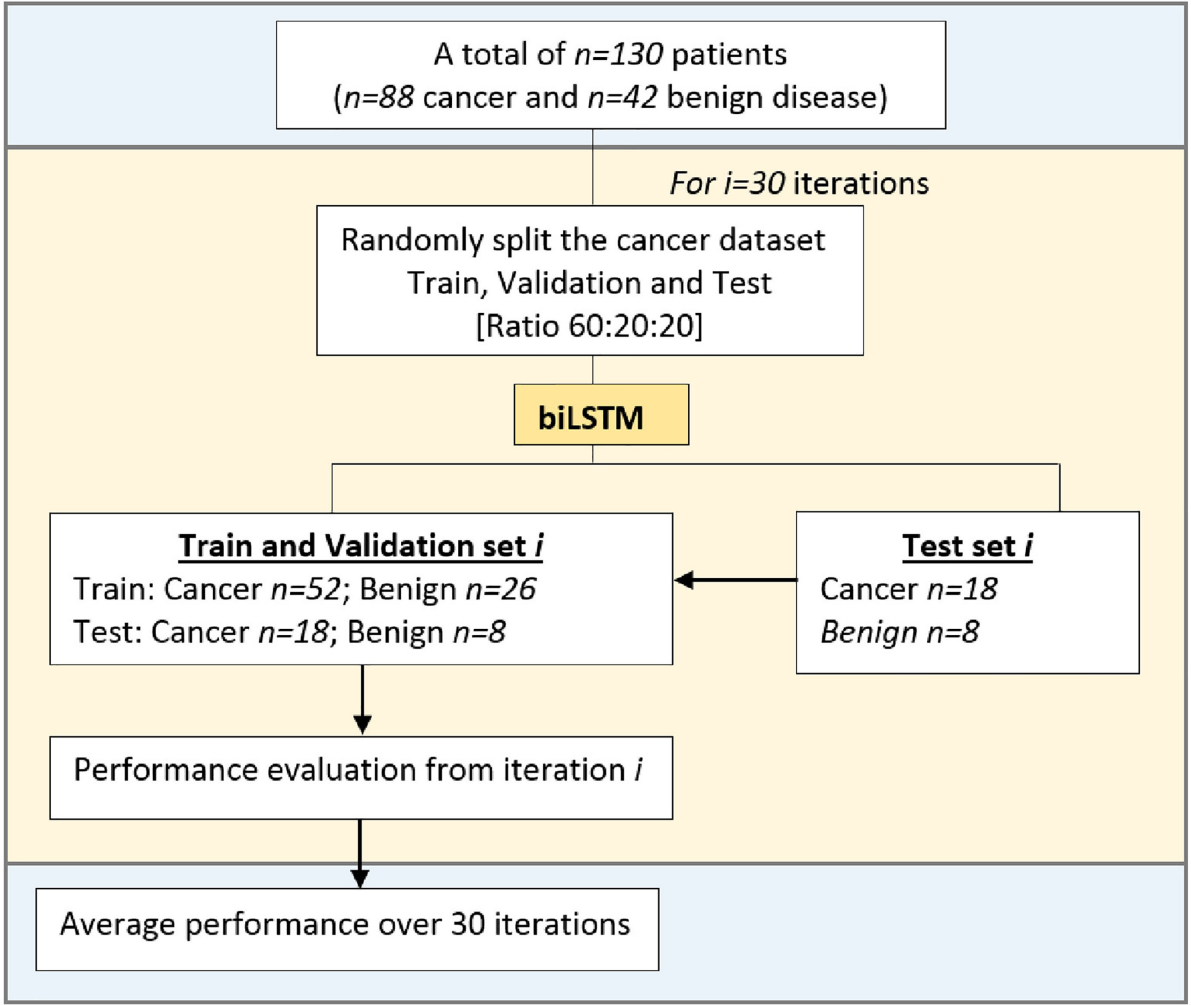
Experimental methodology for evaluating the Deep Learning Models.

The dataset was initially split into datasets derived from men with benign prostate disease and patients with PCa, and each of these datasets was randomly split into ‘train’, ‘validation’ and ‘test’ datasets with a split ratio of 60:20:20, respectively. This random split process was repeated 30 times to create 30 different train, validation and testing sets. This allowed for exhaustive evaluations to be carried out using different sub-populations of the dataset for train, validation, and test purposes. The biLSTM Deep Neural Network models utilised the train sets for training, and the validation sets were utilised during the training process to improve the models’ learning. The test sets are unseen during training, and therefore the test results can be considered to represent mini clinical trials. The results at the end of the 30 runs were collected and analyzed. The methodology for evaluating the Deep Learning models is illustrated in **Figure 3**.

### Performance Evaluation Measures

A set of relevant metrics were adopted for evaluating the performance of the proposed biLSTM models, These were built using six different ‘feature’ sets: FC; PSA; FC+PSA; FC+Age; FC+Age+PSA; Age+PSA. ‘FC’ stands for flow cytometry features and comprises five phenotypic features CD8^+^CD45RA^-^CD27^-^CD28^-^ (*CD8^+^ Effector Memory cells*), CD4^+^CD45RA^-^CD27^-^CD28- (*CD4^+^ Effector Memory cells*), CD4^+^CD45RA^+^CD27^-^CD28^-^ (*CD4^+^ Terminally Differentiated Effector Memory Cells re-expressing CD45RA*), CD3^-^CD19^+^ (*B cells*), CD3^+^CD56^+^CD8^+^CD4^+^ (*NKT cells*), as identified previously as being able to discriminate between benign prostate disease and PCa (11).

Let model *PCaPresence* be a model for detecting the presence of PCa, and *PCaRisk* be a model for predicting whether a patient with PCa has D’Amico high-risk (H-risk) or low/intermediate risk (LI-risk) disease.

- |*TP*| stands for True Positive. |*TP*| in a *PCaPresence* model is the total number of patients diagnosed with PCa who were correctly classified with PCa. |*TP*| in a *PCaRisk* model is the total number of patients diagnosed with H-risk PCa who were correctly classified with H-risk PCa.
- |*TN*| stands for True Negative. |*TN*| in a *PCaPresence* model is the total the number of patients with benign disease who were correctly classified with benign disease. |*TN*| in a *PCaRisk* model is the total the number of LI-risk patients who were correctly classified as LI-risk.
- |*FP*| stands for False Positive. |*FP*| in a *PCaPresence* model is the total number of patients with benign disease who were incorrectly classified with PCa. |*FP*| in a *PCaRisk* model is the total number of LI-risk patients who were incorrectly classified as H-risk.
- |*FN*| stands for False Negative. |*FN*| in a *PCaPresence* model is the total number of patients with PCa who were incorrectly classified with benign disease. |*FN*| in a *PCaRisk* model is the total number of H-risk patients who were incorrectly classified as LI-risk.
- |*P*| stands for Positive. |*P*| in a *PCaPresence* model is the total number of patients with PCa that exist in the dataset. |*P*| in a *PCaRisk* model is the total number of H-risk patients that exist in the dataset. |*P*|=|*TP*|+|*FN*|.
- |*N*| stands for Negative. |*N*| in a *PCaPresence* model is the total number of patients with benign disease that exist in the dataset. |*N*| in a *PCaRisk* model is the total number of LI-risk patients that exist in the dataset. |*N*|=|*FP*|+|*TN*|. The following commonly used evaluation measures can be defined.

(1) $$Accuracy=\frac{\left|TP\right|+\left|TN\right|}{\left|TP\right|+\left|FP\right|+\left|FN\right|+\left|TN\right|},\epsilon \;[0,1]$$

(2) $$Sensitivity=\frac{\left|TP\right|+\left|TN\right|}{\left|TP\right|+\left|FN\right|},\epsilon \;[0,1]$$

Sensitivity is also known as the True Positive Rate (TRP).


(3) $$Specificity=\frac{\left|TN\right|}{\left|TN\right|+\left|FP\right|},\epsilon \;[0,1]$$

Specificity is also known as the True Negative Rate (TNR).


(4) $$FPR=\frac{\left|FP\right|}{\left|FP\right|+\left|TN\right|}=1-Specificity,\epsilon \;[0,1]$$

FPR stands for False Positive Rate.

The closer the values of Accuracy, Sensitivity (i.e. TPR, Sensitivity) and Specificity (i.e. TNR, Specificity) are to 100%, then the better the performance of a model.

The Receiver Operating Characteristic (ROC) evaluates the quality of a prediction model’s performance. The ROC curve has an optimal ROC point which comprises two values: the FPR and the TPR values. The optimal ROC point is computed by function (6) for finding the slope, S.


(5) $$S=\frac{Cost(P\backslash N)-Cost(N\backslash N)}{Cost(N\backslash P)-Cost(P\backslash P)}\times \frac{N}{P},$$

where, in a *PCaPresence* detection model let the positive class be the class containing patients with PCa, and the negative class be the class containing men having benign prostate disease. In the *PCaRisk* prediction model let the positive class be the H-risk group and the negative class be class containing the records of the patients belonging to the low and intermediate class (LI-risk). (N|P) is the cost of misclassifying a positive class as a negative class; and *Cost* (P|N) is the cost of misclassifying a negative class, as a positive class.

The optimal ROC point is identified by moving the straight line with slope *S* from the upper left corner of the ROC plot (FPR=0%, TPR=100%) down and to the right until it intersects the ROC curve. The Area Under the ROC Curve (AUC) is another important performance evaluation metric which reflects the capacity of a model to discriminate between the data obtained from individuals with benign prostate disease and patients with PCa. The larger the AUC, the better the overall capacity of the classification system to correctly distinguish between benign disease and PCa.

### Pre-Processing of Dataset

The dataset comprised 7 features, 5 of which were peripheral blood flow cytometric T and B cell phenotyping features identified in our previous study (11) and the remaining two of which were the clinical features PSA level and Age. The five phenotypic features were: CD8^+^CD45RA^-^CD27^-^CD28^-^ (*CD8^+^ Effector Memory cells*), CD4^+^CD45RA^-^CD27^-^CD28^-^ (*CD4^+^ Effector Memory cells*), CD4^+^CD45RA^+^CD27^-^CD28^-^ (*CD4^+^ Terminally Differentiated Effector Memory Cells re-expressing CD45RA*), CD3^-^CD19^+^ (*B cells*), CD3^+^CD56^+^CD8^+^CD4^+^ (*NKT cells*). The data for each immune phenotyping feature were standardized using z-score transformation. The standardized z-scores are scores (or data values) that have been given a common standard. This standard is a mean of zero and a standard deviation of 1. The PSA and Age values were not standardized (**Table 5**).

**Table 5:** Dataset statistics.

	Benign Prostate Disease				Prostate Cancer			
	Min	Max	Mean	SD	Min	Max	Mean	SD
CD8^+^CD45RA^-^CD27^-^CD28^-^	-0.53	6.02	-0.11	1.01	-0.53	6.02	0.05	0.99
CD4^+^CD45RA^-^CD27^-^CD28^-^	-0.49	4.74	0.10	1.07	-0.49	6.19	-0.05	0.97
CD4^+^CD45RA^+^CD27^-^CD28^-^	-0.40	4.22	0.05	1.00	-0.40	6.02	-0.03	1.00
CD3^-^CD19^+^	-1.03	8.94	0.22	1.50	-0.94	3.10	-0.11	0.62
CD3^+^CD56^+^CD8^+^CD4^+^	-0.37	3.82	0.01	0.68	-0.36	9.93	0.00	1.12
PSA	4.7	19	8.43	3.37	3	2617	51.00	279
Age	51	77	65.88	5.83	52	88	69.88	8.02

The Kolmogorov-Smirnov and Shapiro-Wilk statistical tests demonstrated that the data are not normally distributed and non-parametric tests were therefore used for the analyses (**Table 6**).

**Table 6:** Tests for normal distribution in data.

	Kolmogorov-Smirnova^a^			Shapiro-Wilk		
	Statistic	df	Sig.	Statistic	df	Sig.
CD8^+^CD45RA^-^CD27^-^CD28^-^	0.297	130	0.000	0.526	130	0.000
CD4^+^CD45RA^-^CD27^-^CD28^-^	0.311	130	0.000	0.509	130	0.000
CD4^+^CD45RA^+^CD27^-^CD28^-^	0.346	130	0.000	0.431	130	0.000
CD3^-^CD19^+^	0.195	130	0.000	0.560	130	0.000
CD3^+^CD56^+^CD8^+^CD4^+^	0.354	130	0.000	0.299	130	0.000
PSA	0.441	130	0.000	0.104	130	0.000
Age	0.098	130	0.004	0.979	130	0.037

a. Lilliefors Significance Correction.

Kolmogorov-Smirnov statistic with a Lilliefors significance level for testing normality, and the Shapiro-Wilk statistic. All features have a Sig. value p<0.05 and thus are not normally distributed. For this reason, non-parametric tests were used.

## Results

### Differences in Measured Features Between Men With Benign Prostate Disease and Patients With PCa

The Mann-Whitney U test revealed that there are no significant differences (p<0.05) between the groups for the flow cytometry features, but that the age and PSA levels in men with benign disease and those with PCa were different (p<0.05, **Table 7**). Given that there are 7 comparisons, the Bonferroni correction was applied and the α value was set to α= 0.007 to reduce Type I error. Using the adjusted α value revealed that there were no significant differences between the values of the features of the benign and PCa groups.

**Table 7:** Statistical tests for checking on significant differences between groups.

	Mann-Whitney U	Wilcoxon W	Z	Asympt. Sig (2-tailed)	Effect sizer=Z√N
CD8^+^CD45RA^-^CD27^-^CD28^-^	1700.5	2603.5	-0.734	0.463	0.064 (small)
CD4^+^CD45RA^-^CD27^-^CD28^-^	1614.5	5530.5	-1.162	0.245	0.102 (small)
CD4^+^CD45RA^+^CD27^-^CD28^-^	1559.5	5475.5	-1.436	0.151	0.126 (small)
CD3^-^CD19^+^	1598.0	5514.0	-1.245	0.213	0.109 (small)
CD3^+^CD56^+^CD8^+^CD4^+^	1562.0	5478.0	-1.424	0.154	0.125 (small)
PSA	1302.5	2205.5	-2.717	0.007	0.238 (small)
Age	1344.0	2247.0	-2.515	0.012	0.221 (small)

The alpha value has been set to alpha = 0.007 after the Bonferroni correction. This means that there are no significant differences in the mean values for the benign prostate disease and PCa groups since none of the p values are less than 0.007. The effect size, r values, show that differences between the groups are small and any differences between the benign prostate disease and PCa groups are trivial.

**Table 7** also reports the effect size which is the magnitude of the difference between groups, and it is computed using r=Z√N, where Z is the output of the Mann-Whitney U Test, and N is the total number of samples. According to Cohen (17), the effect size is low if the value of r varies around 0.1, medium if r varies around 0.3, and large if r varies more than 0.5. This means that if the values of two groups do not differ by 0.2 standard deviations or more, then the difference is trivial, even if it is statistically significant. Hence, it can be concluded that there are no statistically significant differences between the features indicated in **Table 7** in the benign prostate disease and PCa groups, and that any differences that do exist are small and trivial.

**Figure 4:**
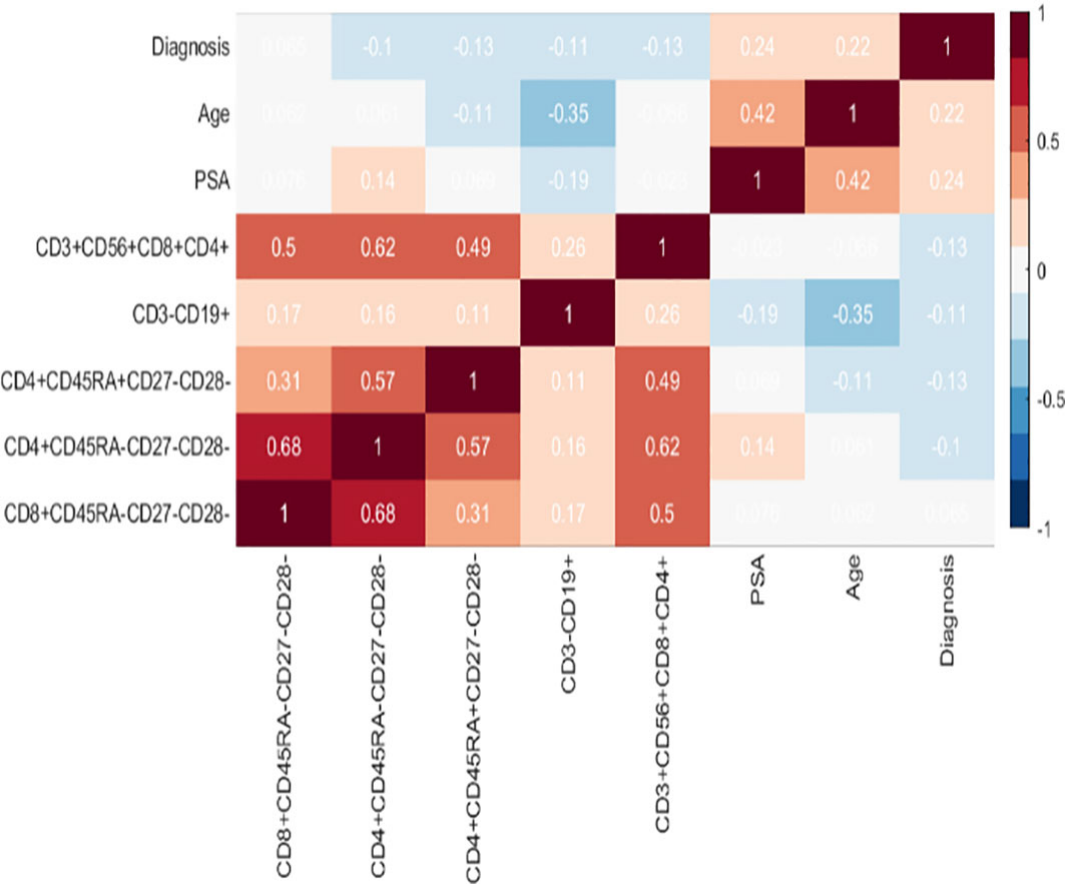
Heatmap of flow cytometry and other features. Each cell of the heatmap provides a Spearman rho correlation value between two features. There are no strong positive or strong negative correlations amongst the inputs.

The nonparametric Spearman’s rank-order correlation shows there to be no strong positive or strong negative correlations amongst the outputs which will be utilised to build the machine learning classifier (**Figure 4**).

### TPTP *vs* TRUS: Differences in Patient Profiles

TPTP is significantly better at diagnosing PCa than TRUS biopsies in biopsy naïve men with an elevated PSA <20 ng/ml and a benign feeling prostate (10). Nafie et al. have therefore proposed that TPTP should be regarded as the biopsy technique of choice in such cases (10).

The Kruskal-Wallis H test is an extension of the Mann-Whitney U test, is the nonparametric equivalent of the one-way analysis of variance and detects differences in distribution location. The major difference between the Mann-Whitney U and the Kruskal-Wallis H is simply that the latter can accommodate more than two groups. Both tests require independent (between-subjects) designs and use summed rank scores to determine the results. Therefore, for the analysis in this subsection the Kruskal-Wallis H test was suitable. The Kruskal-Wallis H (also known as the ‘one-way ANOVA on ranks’) rank-based nonparametric test, was used to determine whether there are any statistically significant differences between the immunophenotypic profiles of the patients when grouped based on biopsy methods and diagnosis. Therefore, a new variable was created, *BiopsyDiagnosis*, where the biopsy type (i.e. TPTP or TRUS) and diagnosis (i.e. benign prostate disease or PCa) were merged into a four separate labels: *TPTPBenign*, *TRUSBenign*, *TPTPCancer*, *TRUSCancer*, and the Kruskal-Wallis test was applied to determine significant difference between the *TPTPBenign* and *TRUSBenign* groups, and between the *TPTPCancer* and *TRUSCancer* patient groups. **Table 8** shows the characteristics of each group of subjects and **Table 9** the results of the Kruskal-Wallis H test which was applied to determine differences between the ranks of the abovementioned groups.

**Table 8:** Patients by biopsy group.

	Frequency	%	Valid %	Cumulative %
*TPTPBenign*	31	23.8	23.8	23.8
*TRUSBenign*	11	8.5	8.5	32.3
*TPTPCancer*	41	31.5	31.5	63.8
*TRUSCancer*	47	36.2	36.2	100.0
**Total**	**130**	**100**	**100**	

**Table 9:** Statistical tests for checking on significant differences between biopsy groups and diagnosis.

	Kruskal-Wallis H	df	Asymp. Sig.
***TPTP Benign vs TRUS Benign***			
CD8^+^CD45RA^-^CD27^-^CD28^-^	0.005	1	0.943
CD4^+^CD45RA^-^CD27^-^CD28^-^	0.000	1	1.000
CD4^+^CD45RA^+^CD27^-^CD28^-^	0.005	1	0.943
CD3^-^CD19^+^	3.511	1	0.061
CD3^+^CD56^+^CD8^+^CD4^+^	0.001	1	0.977
***TPTP Cancer vs TRUS Cancer***			
CD8^+^CD45RA^-^CD27^-^CD28^-^	2.267	1	0.132
CD4^+^CD45RA^-^CD27^-^CD28^-^	3.086	1	0.079
CD4^+^CD45RA^+^CD27^-^CD28^-^	0.373	1	0.541
CD3^-^CD19^+^	5.196	1	0.023
CD3^+^CD56^+^CD8^+^CD4^+^	0.039	1	0.844

Kruskal-Wallis Test.

Grouping variable BiopsyDiagnosis.

The α level for these tests was set to 0.005, however applying a Bonferroni correction which was applied to reduce the chance of a false positive (i.e. a Type I error) reduced the α value to 0.05 since there exist 10 possible comparisons. As shown in **Table 9**, the absence of any significant differences (Asymp. Sig) between any of the immunophenotyping features of the *TPTPBenign* and *TRUSBenign* patients is a good indicator that data collected during TPTP and TRUS biopsy can be combined when training a machine learning model.

### Results of the Deep Learning Models for Identifying the Presence of PCa

The performance of various biLSTM Deep Neural Network models (whose architecture is described above) for predicting the presence of PCa when using six different subsets of features was assessed. **Table 10** shows the training, validation, and test results of the models. **Table 10** shows that the FC+Age was the best model, achieving an accuracy of 86.92% on the validation set, and 86.79% on the test set. More specifically, the model was able to detect the presence of PCa in the validation set with Acc: 86.92% (± 0.10), Sensitivity: 83.70% (± 0.16); Specificity: 94.17% (± 0.11); AUC: 88.94% (± 0.07), ORP-FPR: 9.17% (± 0.20), ORP-TPR: 85.74% (± 0.14) (**Table 10**). Results from the test set (set not used during training or validation) were Acc: 86.79% (± 0.10), Sensitivity: 82.78% (± 0.15); Specificity: 95.83% (± 0.11); AUC: 89.31% (± 0.07), ORP-FPR: 7.50% (± 0.20), ORP-TPR: 84.44% (± 0.14).

**Table 10:** Results of the biLSTM Deep Neural Network models for predicting the presence of PCa.

BILSTM	Training	Validation Results (Mean ± SD)						Test Results (Mean ± SD)					
		Acc	Sens	Spec	AUC	ORP-FPR	ORP-TPR	Acc	Sens	Spec	AUC	ORP-FPR	ORP-TPR
FC	75.9 ± 3.2	65.7 ± 0.2	72.0 ± 0.4	51.7 ± 0.4	61.9 ± 0.2	77.1 ± 0.3	97.6 ± 0.1	66.0 ± 0.2	80.2 ± 0.3	33.8 ± 0.4	57.9 ± 0.1	82.1 ± 0.3	97.0 ± 0.1
PSA	70.5 ± 3.8	85.2 ± 0.1	93.7 ± 0.1	66.3 ± 0.4	80.0 ± 0.2	37.1 ± 0.4	95.6 ± 0.1	88.1 ± 0.1	95.4 ± 0.1	71.7 ± 0.3	83.5 ± 0.1	28.3 ± 0.3	95.4 ± 0.1
FC+PSA	71.7 ± 4.8	82.3 ± 0.1	90.9 ± 0.2	62.9 ± 0.4	76.9 ± 0.2	43.8 ± 0.4	94.8 ± 0.1	83.1 ± 0.1	93.3 ± 0.1	60.0 ± 0.4	76.7 ± 0.2	40.0 ± 0.4	93.3 ± 0.1
**FC+ Age**	**75.6 ± 4.9**	**86.9 ± 0.1**	**83.7 ± 0.2**	**94.7 ± 0.1**	**88.9 ± 0.1**	**9.2 ± 0.2**	**85.7 ± 0.1**	**86.8 ± 0.1**	**82.8 ± 0.2**	**95.8 ± 0.1**	**89.3 ± 0.1**	**7.5 ± 0.2**	**84.4 ± 0.1**
FC+Age+ PSA	78.7 ± 5.8	80.8 ± 0.1	78.9 ± 0.2	85.0 ± 0.2	81.9 ± 0.1	25.0 ± 0.3	85.6 ± 0.2	84.6 ± 0.2	82.2 ± 0.2	90 ± 0.2	86.1 ± 0.1	20.0 ± 0.3	89.4 ± 0.1
Age+PSA	80.0 ± 4.3	86.0 ± 0.1	81.7 ± 0.2	95.8 ± 0.1	88.8 ± 0.1	14.2 ± 0.3	87.6 ± 0.1	82.3 ± 0.1	80.0 ± 0.2	87.5 ± 0.2	83.8 ± 0.1	25.8 ± 0.4	88.9 ± 0.1

The results are the average of 30 iterations when the dataset is split into 60:20:20 ratio corresponding to 60% training set, 20% test set, and 20% validation set. Bold represent the best performing combination.

The validation results for predicting the presence of PCa using PSA revealed a 27.91% lower ORP-FPR when combining FC+PSA than when using PSA alone. For the test results, the ORP-FPR was 20.83% lower when combining FC+PSA than when using PSA alone. The standard deviation values of the FC+PSA were lower indicating a more stable model.

### The Role of Age and Its Impact on Predicting the Presence of PCa

**Figure 5:**
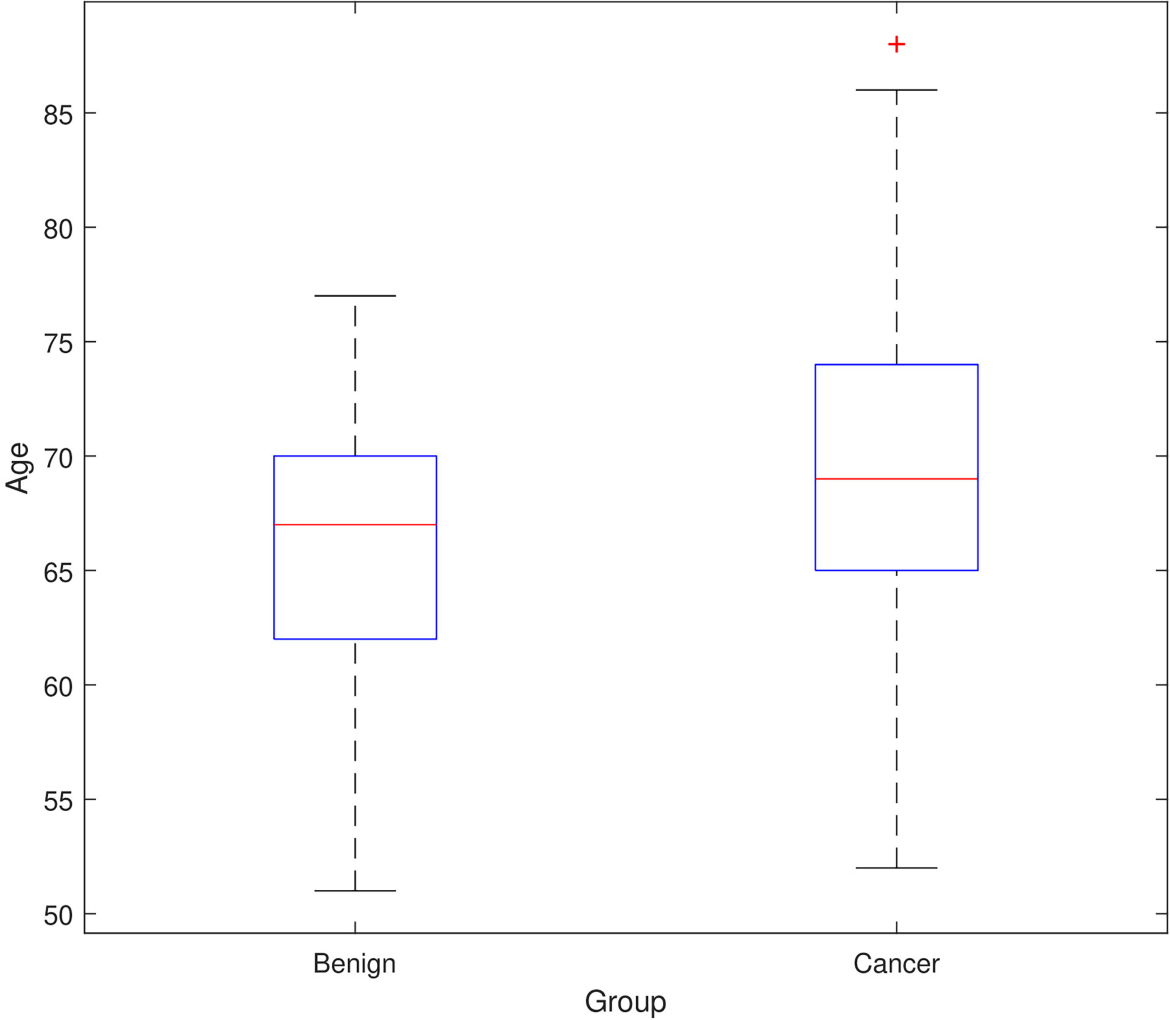
Age of men with benign prostate disease and patients with prostate cancer (PCa).

Combining Age with immunophenotypic features improved prediction accuracy and therefore age appears to be a good predictor for the presence of PCa when combined with the flow cytometry phenotypic features. As the correlation chart in **Figure 4** shows there to be no strong positive or strong negative correlations between age and the rest of the features including diagnosis, we can rule out the fact that correlation is biasing the models’ predictions (i.e. since there are no strong positive or strong negative correlations between Age and the presence of PCa). However, a further statistical investigation was used to conclude whether age is biasing the performance of the prediction models. The two-sample Kolmogorov-Smirnov test, a nonparametric hypothesis test was applied for testing if the variable age has identical distributions in the two populations (i.e. the benign prostate disease and PCa groups). **Note that this is different to the results shown in **Table 6** *which checks whether the variables are normally distributed, and not whether the two groups follow the same distributions.* **Figure 5** shows the distribution of Age values across the benign prostate disease and PCa groups.**

The α value was set to 0.01 to minimize Type I error. The test returned p=0.033, Z=1.431 (and p>0.01) meaning that samples from the benign prostate disease and PCa groups are from the same continuous distribution at the 1% significance level. The next step was to determine whether there are any significant differences in the mean age ranks of these two groups that could be biasing the prediction. The α value was again set to 0.01 to prevent Type I errors and make it harder to predict significant differences. As the Mann-Whitney test revealed p=0.012, Z=-2.515 we can assume that there are no significant differences in the mean ranks of age at the 1% significance level.

However, to further ensure the correct conclusions are reached, the Moses Test of extreme reaction was carried out to recompute the differences between groups when the extreme outliers are not considered. The test is a distribution-free non-parametric test of the difference between two independent groups in the extremity scores (in both directions) that the groups contain. For the benign prostate disease and PCa groups, Moses tests whether extreme values are equally likely in both populations, or if they are more likely to occur in the population from which the sample with the larger range was drawn. The scores from the benign prostate disease and PCa groups are pooled and converted to ranks, and the test statistic is the span of scores (computed as the range plus 1) in one of the groups chosen arbitrarily. An exact probability is computed for the span and then recomputed after dropping a specified number of extreme scores from each end of its range. The exact one-tailed probability is calculated. After trimming the entire dataset, there were 5 patients ≤54 years old and 6 patients ≥84 years old. The information for the Moses extreme reaction test shows that the benign prostate disease and PCa groups have different age values with a Sig. = 0.006. However, when removing the extreme outliers, the Sig. value increases to Sig. = 0.082 hence the two groups have similar age values when the extreme outliers are removed.

Revisiting the results in **Table 7**, and based on the observations described in this section, we can conclude that the algorithms are not biased towards age, and that age in combination with the immunophenotypic features forms a good predictor for the presence of PCa. It is important to mention that if age was biasing the output of the prediction model, then other machine learning models (FC+Age+PSA, Age+PSA) would have consistently delivered high prediction results, because machine learning models excel at detecting patterns in data and would have found the association (pattern) between the Age and the output variable (diagnosis) if this had existed. Based on these observations, it can be concluded that age is not biasing the output of the prediction model.

### Results of the Deep Learning Models for Predicting the Clinical Risk of PCa

Men diagnosed with low-risk or small volume intermediate-risk PCa will very rarely require treatment compared to men who have been diagnosed with high-risk PCa. It is therefore important to detect men in the H-risk group accurately to prioritise treatment for those men, and to prevent unnecessary invasive procedures.

Consequently, we determined whether biLSTM models can differentiate between the clinical risk of PCa using the same features as those which have been used for building the models for predicting the presence of PCa (**Table 11**). Given that there are 85 patients having low-risk (n=18), intermediate-risk (n=44) or high-risk (n=25) PCa, patients were grouped into L/I (low-intermediate) and H (high) risk groups. The biLSTM model that was designed for predicting risk was then utilised to predict risk (L/I or H). The test results in **Table 11** show that the model which combined the flow cytometry features with PSA was able to predict clinical risk in the validation set with Acc: 94.51% (± 6.35), Sensitivity: 92% (± 21.09); Specificity: 95.56% (± 2.99); AUC: 93.78% (± 10.50), ORP-FPR: 4.44% (± 2.99), ORP-TPR: 92% (± 21.09). The results on the test set with Acc: 94.90% (± 6.29), Sensitivity: 92% (± 21.39); Specificity: 96.11% (± 0.00); AUC: 94.06% (± 10.69), ORP-FPR: 3.89% (± 0.00), ORP-TPR: 92% (± 21.39). These are a positive indicator, and it is expected that with a larger dataset the model will be able to learn better, and the standard deviation values will reduce. Comparing the results of the FC+PSA model with those which uses PSA values alone, the FCA+PSA model returned better validation and test results. It therefore appears that PSA is a good predictor of clinical risk when combined with FC values.

**Table 11:** Results of the biLSTM deep neural network models for predicting the D’Amico risk of PCa.

BILSTM	Training	Validation Results (Mean ± SD)						Test Results (Mean ± SD)					
		Acc	Sens	Spec	AUC	ORP-FPR	ORP-TPR	Acc	Sens	Spec	AUC	ORP-FPR	ORP-TPR
FC	78.2 ± 2.2	72.0 ± 17.6	0.71 ± 0.2	0.55 ± 0.4	0.78 ± 0.3	0.66 ± 0.2	0.1 ± 0.1	0.4 ± 0.4	0.7 ± 0.2	0.6 ± 0.4	0.8 ± 0.3	0.7 ± 0.2	0.1 ± 0.1
PSA	91.6 ± 4.3	93.9 ± 6.5	91.3 ± 21.7	95.0 ± 3.0	93.2 ± 10.8	5.0 ± 3.0	91.3 ± 21.7	94.9 ± 7.1	92.7 ± 23.1	95.8 ± 4.5	94.2 ± 11.5	4.2 ± 4.5	92.7 ± 23.1
**FC+PSA**	**90.6 ± 4.4**	**94.5 ± 6.4**	**92.0 ± 21.1**	**95.6 ± 3.0**	**93.8 ± 10.5**	**4.4 ± 3.0**	**92.0 ± 21.1**	**94.9 ± 6.3**	**92.0 ± 21.4**	**96.1 ± 0.0**	**94.1 ± 10.7**	**3.9 ± 0.0**	**92.0 ± 21.4**
FC+ Age	81.0 ± 3.0	86.3 ± 0.0	98.7 ± 0.0	81.1 ± 0.0	89.9 ± 0.0	17.7 ± 0.0	88.7 ± 0.0	87.8 ± 0.0	100.0 ± 0.0	82.8 ± 0.0	91.4 ± 0.0	14.4 ± 0.0	93.3 ± 0.0
FC+Age+ PSA	90.3 ± 5.2	92.9 ± 5.9	88.0 ± 20.1	95.0 ± 0.0	91.5 ± 10.1	5.0 ± 0.0	88.0 ± 20.1	93.1 ± 6.7	83.3 ± 22.7	97.2 ± 0.0	90.3 ± 11.4	2.8 ± 0.0	83.3 ± 22.7
Age+PSA	91.7 ± 3.6	92.8 ± 5.2	87.3 ± 17.7	95.0 ± 0.0	91.2 ± 8.8	5.0 ± 0.0	87.3 ± 17.7	92.6 ± 8.2	85.3 ± 28.0	95.6 ± 0.0	90.4 ± 14.0	4.4 ± 0.0	85.3 ± 28.0

The results are the average of 30 iterations when the dataset is split into 60:20:20 ratio corresponding to 60% training set, 20% test set, and 20% validation set. Bold represent the best performing combination.

Experimental results described above using models for detecting the presence of PCa found that Age is a feature which, when combined with the immunophenotypic profiling, delivers a greater predictive accuracy than when it is used alone. Here, we follow a similar analysis for interrogating the impact of PSA. As indicated in the correlation chart (**Figure 4**), there are no strong positive or strong negative correlations between PSA and the other features.

The two-sample Kolmogorov-Smirnov test was applied to check for identical distributions in the two populations (i.e. benign prostate disease and PCa). The test returned p=0.033, Z=1.431 (and p>0.01) meaning that samples from the benign prostate disease and the PCa groups are of the same continuous distribution at the 1% significance level. Next, we determined whether significant differences in the mean ranks of the benign prostate disease and PCa groups could be biasing the prediction. The alpha value was set to 0.01 to prevent Type I errors and make it harder to predict significant differences. The Mann-Whitney test returned p=0.007, Z=-2.717. Therefore, it can be assumed that there are significant differences in the mean ranks of age at the 1% significance level. These results show that the PSA could be influencing the risk of disease, which makes clinical sense given that the high-risk patients often (but not always) have higher PSA values than the low and intermediate-risk patients (**Table 11**).

## Discussion

It is essential that men with low-risk prostate abnormalities are not diagnosed with PCa, as those with low-grade disease do not require active treatment, yet they become `labelled’ as having PCa. This can have adverse psychological and financial consequences and assign these men to life-long surveillance. Inappropriate assignment of men to potentially life-threatening invasive procedures and lifelong surveillance for PCa has significant psychological, quality of life, financial and societal consequences. Although the diagnosis of PCa based on PSA levels alone is not reliable, combining PSA measurements with other approaches might strengthen the diagnostic value of PSA measurements and identifying its clinical risk, and it is based on this concept that the current study has been performed.

Given the established reciprocal relationship(s) between cancers and the immune system, we have previously demonstrated (11) that a set of five phenotypic features (CD8^+^CD45RA^-^CD27^-^CD28^-^ (*CD8^+^ Effector Memory cells*), CD4^+^CD45RA^-^CD27^-^CD28^-^ (*CD4^+^ Effector Memory cells*), CD4^+^CD45RA^+^CD27^-^CD28^-^ (*CD4^+^ Terminally Differentiated Effector Memory Cells re-expressing CD45RA*), CD3^-^CD19^+^ (*B cells*), CD3^+^CD56^+^CD8^+^CD4^+^(*NKT cells*) could be used to identify the presence of PCa in a population of asymptomatic men with PSA levels that were elevated above the normal, but <20 ng/ml (‘normal’ is ~5 ng/ml), a population which presents a significant clinical challenge. In a subsequent study we identified an NK cell phenotypic signature which can be used to identify both the presence and clinical risk of PCa in the same cohort of asymptomatic men (12).

Herein, we explored whether this T and B cell phenotypic signature can be incorporated into models that can predict the presence and ***clinical risk*** of PCa in men having elevated PSA values of any level, and whose disease status had been defined using the TRUS and TPTP biopsy. Given limited sample numbers, it was not possible to undertake a similar analysis using the NK cell phenotyping dataset. For this, we built two prediction models: the first to detect the presence of PCa and the second to predict the clinical risk of any PCa present in asymptomatic men with raised PSA values, not just those < 20 ng/ml. Although this signature alone was not suitable for detecting the presence of PCa or its clinical risk in a population of men having PSA values <20 ng/ml, this T and B cell phenotypic signature can be used to build highly accurate machine learning models for predicting the presence of PCa (when combined with Age) and the clinical risk of any PCa which is present (when combined with PSA levels).

Using a set of immunophenotyping biomarkers combined with basic clinical data we have shown it to be possible to develop machine learning models which can predict the presence of PCa and its clinical significance, without the need for invasive biopsies. Inserting the data derived from the analysis of the peripheral blood from an individual into the proposed tool will return a prediction about that individual. The proposed models are based on machine learning methods which can be continually retrained as more patient data are collected to learn patterns from a larger population - this will further increase performance. We expect that the proposed approaches will spare men with benign prostate disease or low-risk PCa from unnecessary invasive diagnostic procedures such as TRUS or TPTP biopsy. We expect that these new approaches could avoid up to 70% of prostate biopsies, thereby sparing men with benign disease or low-risk PCa from unnecessary biopsy and significantly reduce under- and over-diagnosis.

## Data Availability Statement

The datasets presented in this study can be found in online repositories. The names of the repository/repositories and accession number(s) can be found below: Mendeley Data: doi: 10.17632/wmgtzw2w8f.1 (18).

## Ethics Statement

Research Protocols were registered and approved by the National Research Ethics Service Committee of East Midlands and by the Research and Development Department in the University Hospitals of Leicester NHS Trust. All participants were given information sheets explaining the nature of the study and all provided informed consent. Ethical approval for the collection and use of samples from the TPTP cohort (Project Title: Defining the role of Transperineal Template-guided prostate biopsy) was given by NRES Committee East Midlands – Derby 1 (NREC Reference number: 11/EM/3012; UHL11068). Ethical approval for the collection and use of samples from the TRUS cohort (Project title: A pilot study to identify gene fusions in Prostate Cancer) was given by NRES Committee East Midlands – Derby 2 (NREC Reference number: 09/H0401/92; UHL 10856). The patients/participants provided their written informed consent to participate in this study.

## Author Contributions

GC computationally analyzed the flow cytometry data, prepared and tested the algorithms, analyzed the results, wrote the first draft, and made a significant contribution to the preparation of the manuscript. SM contributed to the preparation, staining and analysis of the flow cytometry data, and generated the multidimensional flow cytometry datasets on which the study has been based. SR, GF, CJ, and SH contributed to the preparation, staining, and analysis of the flow cytometry data, and generated the multidimensional flow cytometry datasets on which the study has been based. MK identified the clinical need, provided access to clinical samples and clinical data, and made a significant contribution to the preparation of the manuscript. AP conceived the study and made a significant contribution to the interpretation of the data and the preparation of the manuscript. All authors contributed to the article and approved the submitted version.

## Funding

The authors acknowledge the financial support of the John and Lucille van Geest Foundation and the Healthcare and Bioscience iNet, an ERDF funded initiative managed by Medilink East Midlands. GC acknowledges the financial support of The Leverhulme Trust (Research Project Grant RPG-2016-252). The funders had no role in study design, data collection and analysis, decision to publish, or preparation of the manuscript.

## Conflict of Interest

The authors declare that the research was conducted in the absence of any commercial or financial relationships that could be construed as a potential conflict of interest.

## Publisher’s Note

All claims expressed in this article are solely those of the authors and do not necessarily represent those of their affiliated organizations, or those of the publisher, the editors and the reviewers. Any product that may be evaluated in this article, or claim that may be made by its manufacturer, is not guaranteed or endorsed by the publisher.
